# Comparison of outcomes between medical and surgical treatment in dogs with prostatic adenocarcinoma: a retrospective study

**DOI:** 10.1186/s12917-021-03103-5

**Published:** 2022-01-15

**Authors:** Keigo Iizuka, Kumiko Ishigaki, Mamiko Seki, Takahiro Nagumo, Kei Tamura, Naoki Sakurai, Kazuyuki Terai, Kazushi Asano

**Affiliations:** Laboratory of Veterinary Surgery, Department of Veterinary Medicine, College of Bioresource Sciences, Nihon University, 1866 Kameino, Fujisawa, Kanagawa 252-0880 Japan

**Keywords:** Dog, Prostatic adenocarcinoma, Total prostatectomy, Total Prostatocystectomy

## Abstract

**Background:**

Prostatic cancer is uncommon in dogs. Dogs with prostatic carcinoma have been reported to have a poor prognosis. Information regarding prognosis with various surgery options as well as prognosis with surgical vs. medical treatment is lacking. This retrospective study compares the outcomes of medical management to surgical treatment in dogs with prostatic adenocarcinoma and assesses the surgical outcomes of patients who underwent total prostatectomy (TP) and prostatocystectomy (TPC). The medical records of 41 dogs with prostatic adenocarcinoma, between February 2008 and June 2019, were reviewed for information on signalment, clinical signs in the initial evaluation, preoperative diagnostic imaging findings, treatment type (non-surgical or surgical), surgery type, postoperative complications, adjunctive medical therapy, and survival time. The dogs were divided into non-surgical (*n* = 12) or surgical (*n* = 29) groups. The surgical group was subdivided into the TP (*n* = 20) and TPC (*n* = 9) subgroups.

**Results:**

Age was not significantly different between the surgical (median 13.1 years [8.4–15.4] years) and the non-surgical groups (median 10.8 [7.7–15.3] years). Body weight (BW) was also not significantly different between the surgical (median 6.8 kg [2.4–34.5 kg]) and non-surgical groups (median 6.4 kg [3.7–9.12 kg]). The overall median survival time (MST) from the initial evaluation was significantly longer in the surgical than in the non-surgical group (337 vs. 90.5 days). The postoperative MST was significantly longer in the TP group than in the TPC subgroup (510 vs. 83 days). As TPC was performed in cases of tumor progression, its postoperative complications were severe, resulting in a shorter MST. Ten (50%) and 6 patients (30%) in the TP subgroup postoperatively showed mild and severe urinary incontinence, respectively, whereas all patients in TPC subgroup did show severe incontinence.

**Conclusion:**

Results of the study suggest that surgical treatment of prostatic carcinoma results in longer survival times over medical management alone. In particular, TP might be recommended for improving survival time and quality of life in canine prostatic adenocarcinoma that does not infiltrate the bladder. Early detection is key for a survival advantage with surgical treatment.

## Background

Prostatic tumors are relatively uncommon in dogs; they are generally malignant, with adenocarcinoma being the most common type of tumor [1, 2]. Canine prostatic tumors often remain undetected until related clinical signs including stranguria, dysuria, dyschezia, diarrhea, and pain [3, 4]. At the time of diagnosis, canine prostatic carcinoma generally invades surrounding tissues, with a high propensity for regional (30%) and distant metastases (50%) [4–6]. Therefore, canine patients with prostatic carcinoma have been reported to have a poor prognosis (MST; 0–6.9 months) [6, 7].

Cyclooxygenase-2 overexpression has been demonstrated in canine prostatic carcinomas, and treatment with non-steroidal anti-inflammatory drugs (NSAIDs) has demonstrated survival benefits in canines [7]. Previous studies have demonstrated prolonged median survival time (MST) for dogs with prostatic carcinomas treated with a combination of chemotherapy and NSAIDs (MST; 155 days), as opposed to NSAIDs alone (MST; 106 days) [8, 9]. In addition, 3 dogs treated with metronomic chemotherapy, toceranib, and NSAIDs did not show any survival benefit compared to those treated with NSAIDs alone [9]. Furthermore, the effect of tyrosine kinase inhibitors on canine prostatic adenocarcinoma has not yet been reported. To our knowledge, there have been no studies comparing outcomes between medical and surgical treatments.

Surgical treatment is generally recommended for early-stage canine prostatic cancer and intracapsular disease, but case selection being likely to be important for a good outcome [10]. However, surgical treatment is associated with serious postoperative complications, including urinary incontinence, which impairs the quality of life (QOL) [11–13]. Subtotal intracapsular prostatectomy has been demonstrated to have superior outcomes to total prostatectomy (TP), in terms of postoperative survival (MST; 130 days) and incidence of serious complications [14]. A recent study described that TP, combined with adjunct therapies, prolonged survival, and lowered the complication rates in 25 dogs with prostatic cancers, including 8 with adenocarcinomas [15]. However, it remains unclear whether TP is a definitive therapeutic option for canine prostatic adenocarcinomas, because of the small sample size studied to date. In addition, total prostatocystectomy (TPC) may be chosen in cases of tumor invasion into the bladder; however, postoperative urinary tract infection and urinary incontinence are major complications [16]. There is limited information regarding the survival time, QOL, and postoperative complications associated with surgical treatment, including TP and TPC.

The purposes of this retrospective study were to compare the outcomes of medical management and surgical treatment in dogs with prostatic adenocarcinoma and to assess the surgical outcomes of TP and TPC.

## Methods

### Patients

Canine patients diagnosed with prostatic adenocarcinoma at the Animal Medical Center of Nihon University between February 2008 and June 2019 were included in this study. Patients who did not receive therapeutic regimens after the diagnosis of prostatic adenocarcinoma or those who underwent cytology only for the diagnosis of prostatic adenocarcinoma were excluded from this study. Informed owner consent was obtained prior to the inclusion of all canine patients.

In all cases, physical examination, complete blood test, serum chemistry, abdominal ultrasound, and thoracic and abdominal radiography and CT were performed. The diagnosis was made based on the cytopathology of a cell block preparation obtained via ultrasound-guided aspiration of the prostate via the urinary catheter. In the preoperative CT, intracapsular or extracapsular types based on the shape of the prostate margin (smooth or irregular) and extension into the urethra and bladder neck were evaluated in each patient. In addition, potential metastases of the lungs, lymph nodes, and/or bone were evaluated. The prostatic volume was measured on CT images, and the ratio (prostatic volume index) of the prostatic volume to BW was calculated. After surgery, histopathological diagnosis was performed in all cases in the surgical group.

Inclusive owners opted for either medical or surgical treatment after the diagnosis. Canine patients who underwent medical treatment and those who underwent surgical treatment were classified into non-surgical and surgical groups, respectively. Furthermore, the surgical group was divided into TP and TPC subgroups according to the surgical procedure performed.

### Non-surgical group

Canine patients in the non-surgical group were prescribed NSAIDs, such as firocoxib, piroxicam, meloxicam, or carprofen, and/or administration of toceranib.

### Surgical group

One surgeon performed all surgeries and selected the procedure during surgery; TP was chosen when the bladder was judged to be unaffected based on CT finding, whereas TPC was performed when the tumor appeared to have infiltrated the bladder and/or ureter.

Subcutaneous injections of 1.0 mg/kg prednisolone (Kyoritsu Seiyaku Co., Tokyo, Japan), 1.0 mg/kg famotidine (LTL Pharma Co., Tokyo, Japan), and 1.0 mg/kg maropitant citrate (Cerenia®; Zoetis, Troy Hills, NJ, USA) were given. Subcutaneous injection of 0.04 mg/kg atropine sulfate (Mitsubishi Tanabe Pharma Co., Osaka, Japan), followed by intravenous injection of 0.1 mg/kg midazolam (Dormicum; Astellas Pharma Inc., Tokyo, Japan) and 0.1 ml/kg fentanyl citrate-droperidol (Thalamonal; Daiichi-Sankyo Propharma Co., Ltd., Tokyo, Japan) were given for premedication. General anesthesia was induced with propofol (Mylan; Mylan Seiyaku Ltd., Tokyo, Japan). After induction, endotracheal intubation was subsequently performed. Each dog was mechanically ventilated with a mixture of isoflurane and oxygen. For intra- and postoperative medical management, dopamine (1.25–10 μg/kg/min) (Teva Takeda Pharma Ltd., Nagoya, Japan), dobutamine (1.25–10 μg/kg/min) (Kyowa Pharmaceutical Industry Co. Ltd., Osaka, Japan), and nafamostat (0.1–0.2 mg/kg/h) (serine protease inhibitor; Nichi-Iko Pharmaceutical Co. Ltd., Toyama, Japan) were continuously infused. For analgesia, intra- and post-operative continuous drip infusions of remifentanil (5–40 μg/kg/h) and lidocaine (25–75 μg/kg/min) (Aspen Japan Ltd., Tokyo, Japan), and pre- and post-operative intramuscular injections of morphine (0.3 mg/kg each dose) were used.

**Fig. 1:**
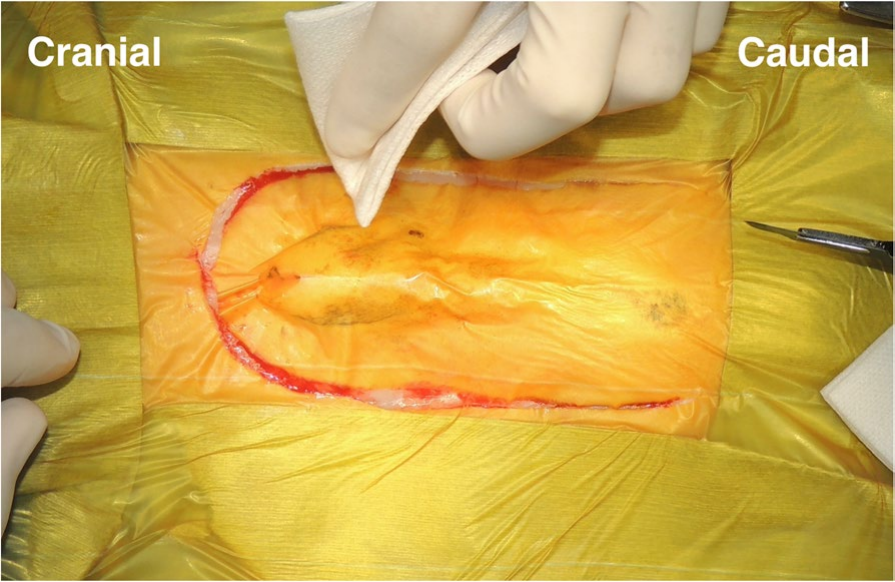
Peri-preputial U-shaped skin incision. In male dogs, an abdominal midline incision may not provide a wide operative field because of the centrally located penis. This incision is indicated for surgery in the male lower urinary tract

**Fig. 2:**
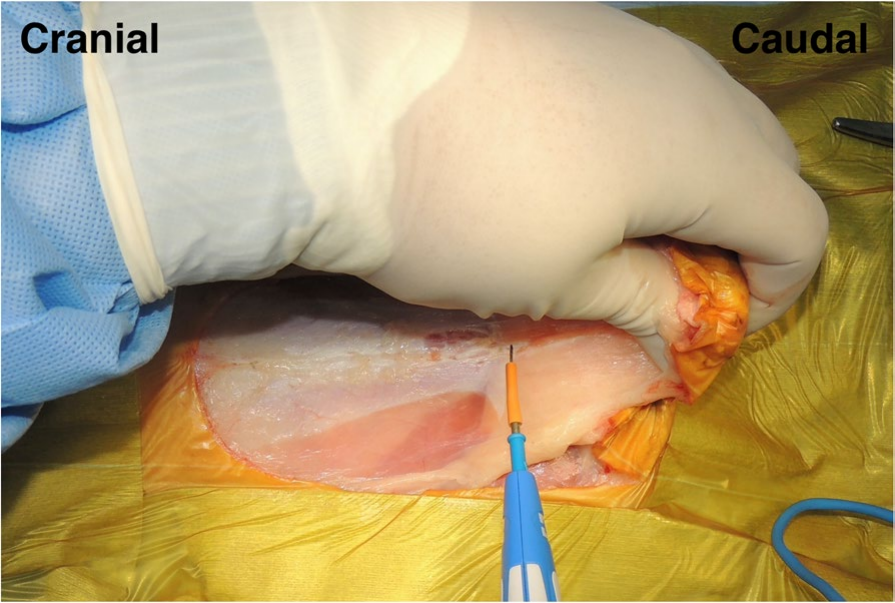
Intraoperative findings after peri-prepuce U-shaped skin incision. A penis was turned over in caudal direction by the blunt dissection after the skin incision

**Fig. 3:**
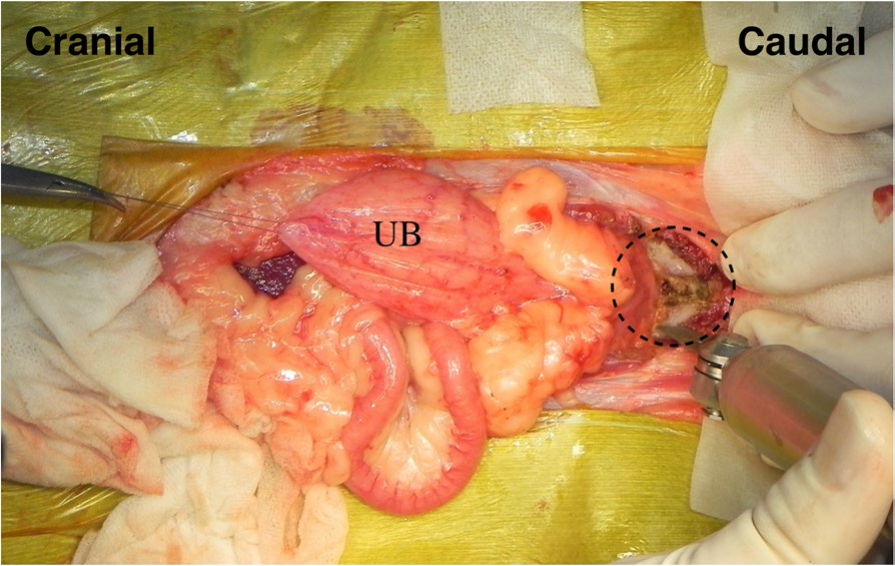
Removal of the cranial part of pubic bone. The bilateral rectus abdominus muscles were dissected from their insertion at the pubis, then the cranial part of the pubic bone was resected by a surgical saw. UB; Urinary Bladder, black dotted line; the cranial part of the pubic bone

Each patient in the surgical group was positioned in dorsal recumbency under general anesthesia. A peri-preputial U-shaped skin incision was made to secure a wide surgical field (Fig. 1). An additional abdominal midline skin incision in the cranial direction was performed when needed. After the penis was turned over by blunt dissection (Fig. 2), caudal celiotomy was performed from the umbilicus to the pubis. In addition, the bilateral rectus abdominis muscles were dissected from their insertion at the pubis border to expose the ureters, bladder, prostate, and urethra. When exposure of the urethra distal to the prostate was insufficient, the cranial part of the pubic bone was partially resected with a surgical saw (Fig. 3). Subsequently, TP or TPC was performed.

**Fig. 4:**
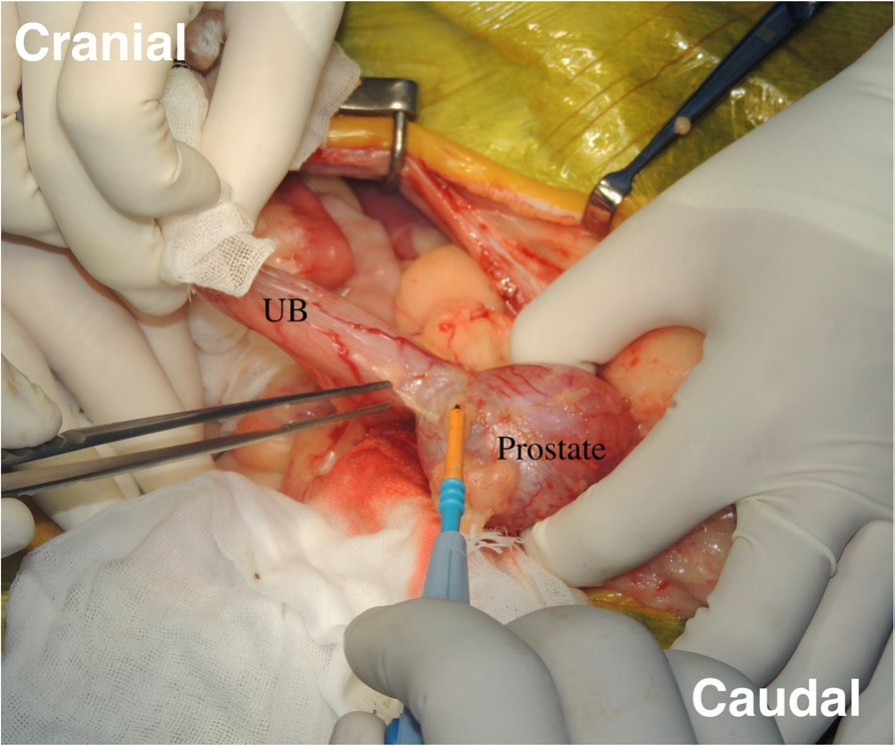
Dissection of the bladder neck and prostate. The border between the bladder neck and prostate was carefully dissected to the vesicourethral junction using electrocautery and was transected by Mayo or Freeman scissors

In the TP procedure, the enlarged prostate was isolated from the surrounding tissues. The bilateral membranous pedicle, including vessels and nerves dorsolateral to prostate, were carefully resected close the prostate by the bipolar electrocautery and/or a vessel sealing system. In particular, the caudal vesical arteries and the nerves running to the bladder from the pelvic plexus were visually recognized and preserved while the prostate was isolated by the dissection. The border between the bladder neck and prostate was carefully dissected to the vesicourethral junction using electrocautery (Fig. 4) and transected using Mayo or Freeman scissors. The pelvic urethra distal to the prostate was dissected using electrocautery, and the urethra was ligated. A stay suture was placed in the urethra distal to the ligation, and the urethra was transected between the ligation and the stay suture. The urinary catheter was inserted from the penis through the urethra in the excision sites in the pelvic urethra and the bladder neck and then onwards into the bladder. Then, the urethral and bladder ends were anastomosed with a simple interrupted pattern using 4–0 or 5–0 long-term absorbable sutures (PDS II®; Johnson & Johnson, New Brunswick, NJ, USA). A parachute suture was applied for vesicourethral anastomosis [17]. In brief, a one-layer simple interrupted suture was firstly placed at the most dorsal side, but not being tied. Second suture was placed at the most ventral side approximately 180 degrees from the first, but not being tied. Third and fourth sutures were placed at the bilateral sides approximately 90 degrees from the first, but not being tied. Additional 2 to 3 sutures were placed to fill the space between each placed suture, but not being tied. After a total 12 to 16 sutures were placed in the entire circumference, each suture was tied for the vesicourethral anastomosis.

**Fig. 5:**
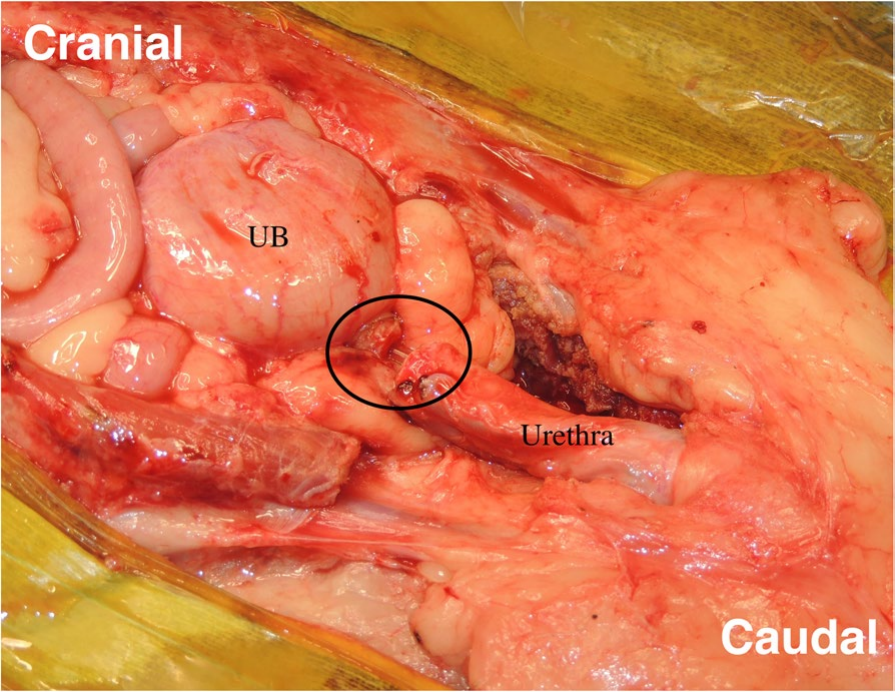
Anastomosis between the bladder and the cavernous portion of the urethra. In case of removal of the urethral membranous portion along with the prostate mass, the bladder was anastomosed to the urethral cavernous portion through the site where pubic bone was originally removed. UB; urinary bladder, black circle; anastomosis site of bladder and urethra

When the pelvic urethra was removed due to extensive tumor infiltration, the bladder was anastomosed to the penile urethra through the original site of the removed pubic bone (Fig. 5). After anastomosis, the abdominal incision was routinely closed, and the caudal part of abdominal wall was sutured with the cavernous portion of penis passing through the wall. The peri-preputial U-shaped skin incision was routinely closed after subcutaneous placement of an active drainage system.

**Fig. 6:**
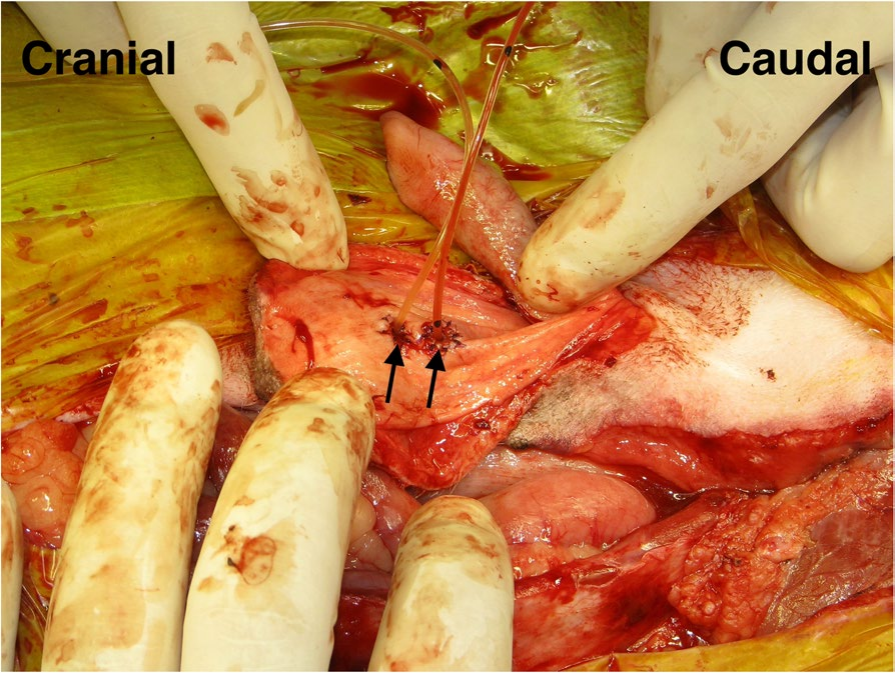
Individual uretero-prepuce mucosal anastomosis after total prostatocystectomy. Each ureter was anastomosed into the prepuce mucosa individually with 5–0 absorbable monofilament suture in a simple interrupted pattern. Black arrow; anastomosis site of ureter and prepuce mucosa

**Fig. 7:**
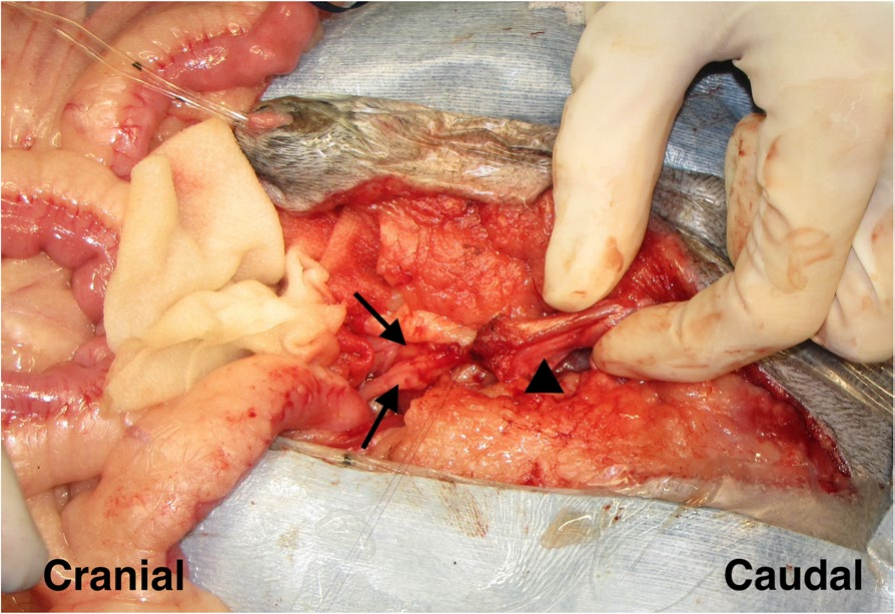
Uretero-urethral anastomosis after total prostatocystectomy. A ureter was anastomosed to another ureter with a functional anastomosis manner, and then the fused ureter was anastomosed to the penile urethra like a Y figure with 5–0 absorbable monofilament suture in a simple interrupted pattern. Black arrows; ureter, black arrowhead; penile urethra

**Fig. 8:**
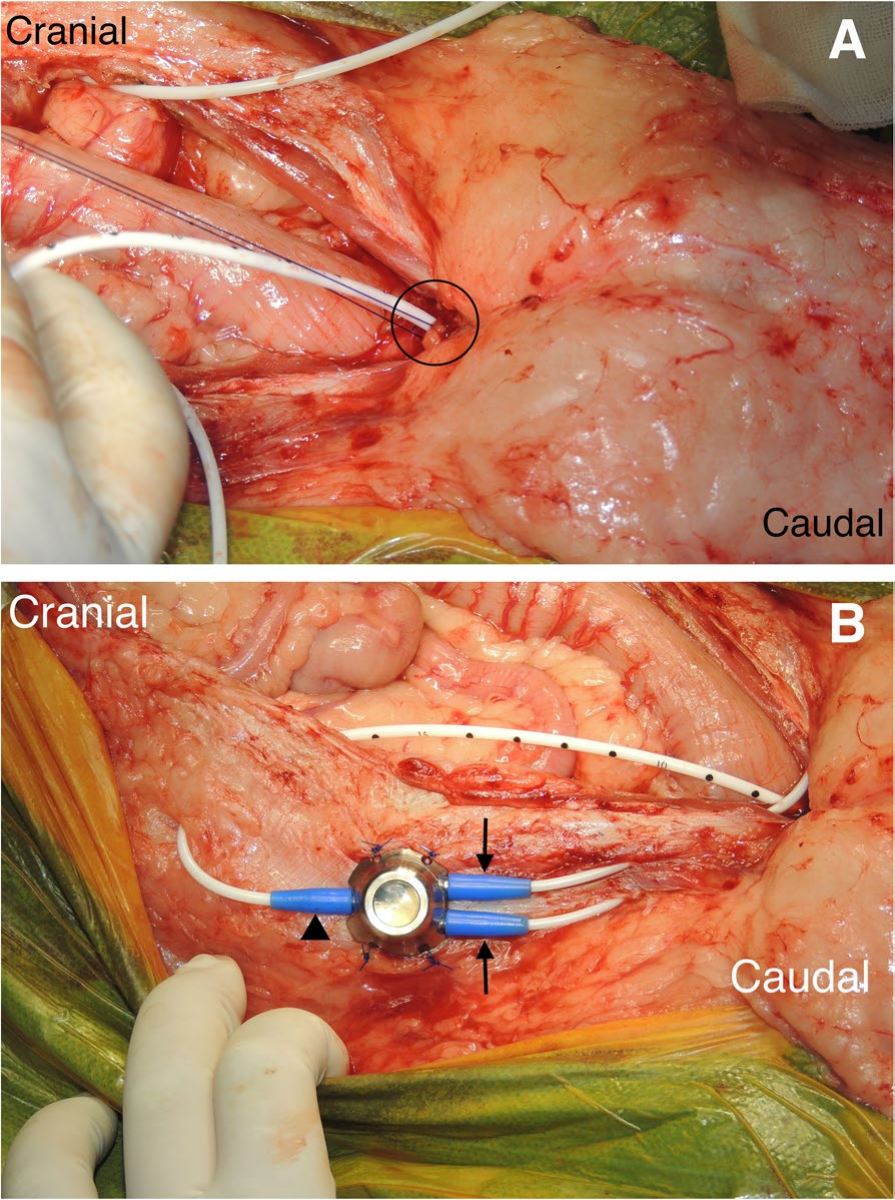
Subcutaneous ureteral bypass system between kidney and urethra after total prostatocystectomy. (**A**) A bladder catheter was connected to the stump of urethra. Black circle; the connection of the bladder catheter to the stump of urethra. (**B**) Two nephrostomy catheters were merged by one pantport. Black arrows; nephrostomy catheters, black arrowhead; black catheter

**Fig. 9:**
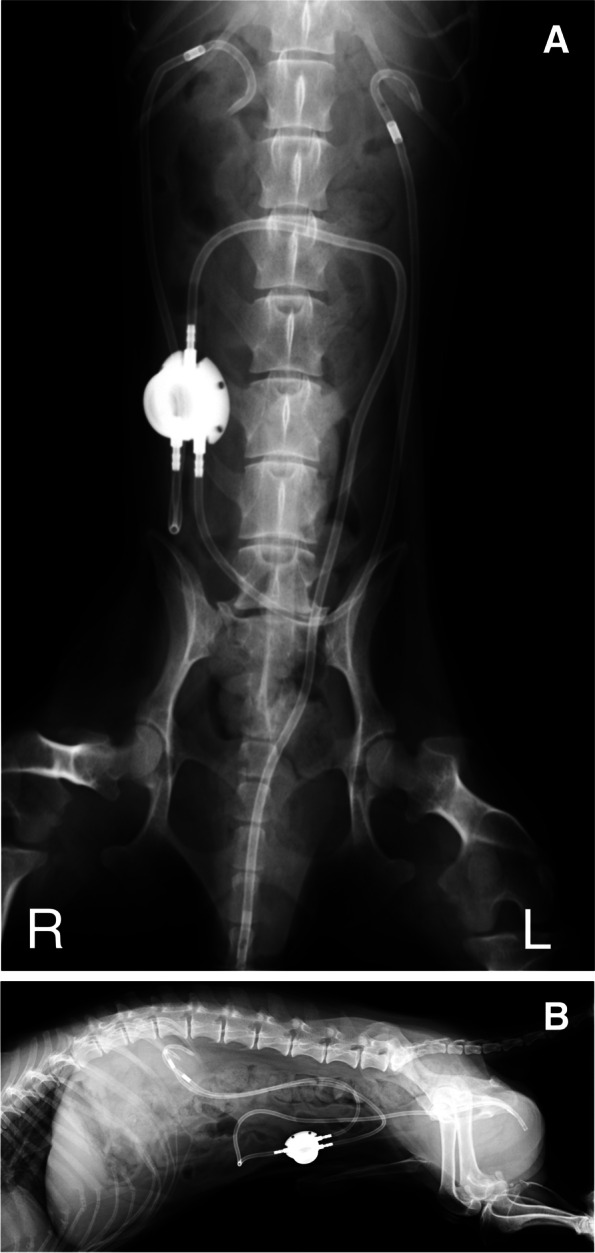
Postoperative radiographs of subcutaneous ureteral bypass system between kidney and urethra with one pantport. (**A**) Ventrodorsal image. (**B**) Lateral image

In the TPC procedure, a U-shaped skin incision was made, similar to the TP. En bloc resection of the bladder and the prostate was completed, and the following 3 different reconstructive patterns were performed: (i) each ureter was anastomosed into the prepuce individually [18] (Fig. 6); (ii) 1 duct was made by suturing the ends of both ureters and this was anastomosed to the urethra [16] (Fig. 7); (iii) the urinary tract was connected to the prepuce or the end of the urethra via a subcutaneous ureteral bypass system (SUB™1.0; SUB1001K, Norfolk VET PRODUCTS, Skokie, IL, USA) [19] (Fig. 8). In the cases of SUB system placement, two options were performed: one was the procedure that 2 nephrostomy catheters and individual ports were used, and another was that 2 nephrostomy catheters were merged by 1 pantport. In the 2 ports, one bladder catheter was placed into the prepuce, and another one was connected into the urethra. In the one pantport, one bladder catheter was inserted into the stump of urethra (Fig. 9). A ureter stent was used to prevent ureteral orifice stricture, where needed. When the lymph nodes were enlarged, lymphadenectomy was performed, similar to TP. After urinary diversion, the abdominal wall was routinely closed. The skin around the penis and prepuce were returned to the original position. A J-VAC™ closed wound drainage system (Johnson & Johnson) was used to actively discharge the subcutaneous transudates. A blake drain was subcutaneously placed and connected with a reservoir. After the placement of the drainage system, 2 subcutaneous sutures were placed in the midline under the prepuce to limit the subcutaneous space. The resected bilateral preputial muscles were apposed with a simple suture pattern using absorbable materials. The skin closure was routinely performed.

### Clinical progress

In the non-surgical group, the survival time from the initial evaluation was recorded for each case. In the surgical group, a histopathological diagnosis of the resected masses was performed. Postoperative complications, including urinary incontinence, were evaluated in each case as non, mild, or severe incontinence, based on the owner’s report. Mild incontinence was determined as being held by the owner, abdominal pressure increase caused by excitement, bedwetting during sleep, and voluntary micturition. Continuous urinary dripping with involuntary micturition was considered as severe incontinence. In addition, the incidence of pyelonephritis after surgery was evaluated.

The survival periods from the initial evaluation and after the operation were recorded.

### Statistical analyses

The numeric data are shown as medians (ranges). The Mann–Whitney U test was used to compare age, BW, hematology and serum chemistry examination parameters, and CT measurements (prostate volume and its index) between the surgical and non-surgical groups. Breed, clinical signs, castration status, CT findings (intra- versus extracapsular type, presence versus absence of invasion into the bladder/urethra, and presence versus absence of lung metastasis) in the surgical and non-surgical groups were compared using the chi-square test. The survival time was analyzed using the log-rank test, and Kaplan–Meier survival curves were constructed between the surgical and non-surgical groups, and between the TP and TPC groups. For all statistical tests, statistical significance was set at *P* < 0.05.

## Results

Forty-one dogs were included in this study and were categorized into surgical (*n* = 29) and non-surgical groups (*n* = 12). All patients in the surgical group were diagnosed with prostatic adenocarcinoma by histopathological evaluation of the resected mass, which corresponded with the cytopathological evaluation of the cell block preparation from ultrasound-guided aspiration of the prostate via a urinary catheter. Age was not significantly different between the surgical (13.1 years [8.4–15.4] years) and the non-surgical groups (10.8 [7.7–15.3 year-old] years) (*P* = 0.206). Body weight (BW) was also not significantly different between the surgical (6.8 kg [2.4–34.5 kg]) and non-surgical groups (6.4 kg [3.7–9.12 kg]) (*P* = 0.224). Small-breed dogs accounted for most of the patients in this study: 22 dogs were Miniature Dachshunds (46%), 3 each were Beagles, mixed breeds, and Pomeranians, and 2 were Papillons. The remaining eight breeds were American cocker spaniel, Boston terrier, Chihuahua, Italian greyhound, Jack Russel terrier, Maltese, Miniature Schnauzer, Shetland sheepdog, and Shih-Tzu. The ratio of miniature dachshunds was significantly lower in the surgical group (12/29 cases, 41%) than in the non-surgical group (10/12 cases, 83%) (*P* = 0.008). Forty dogs had already been castrated, and the castration status was significantly different between the surgical (29/29 cases, 100%) and non-surgical groups (9/12 cases, 75%) (*P* = 0.005). Thirty-seven patients (37/41 cases, 90%) showed clinical signs, including dysuria (21/41 cases, 51%), hematuria (21/41 cases, 51%), and dyschezia (20/41 cases, 48%), in the initial evaluation. The dysuria and dyschezia ratios were not significantly different between the groups (dysuria, 15/29 cases; 52% vs. 6/12 cases, 50%; dyschezia: 14/29 cases, 48% vs. 6/12 cases, 50% [surgical vs. non-surgical group]). However, hematuria differed between the groups (18/29 cases, 62% vs. 3/12 cases, 25% [surgical vs. non-surgical group]) (*P* = 0.031).

The hematological and serum chemistry examinations are summarized in Table 1. In the hematology examination, there were no significant differences between the surgical and non-surgical groups. In the serum chemistry examinations, there were no significant differences between the surgical and non-surgical groups except for the creatinine levels (reference range 0.5–1.8 mg/dL), which were significantly higher in the surgical group (0.9 mg/dL [0.4–1.6 mg/dL]) than in the non-surgical group (0.6 mg/dL [0.2–0.8 mg/dL]) (*P* = 0.001).

**Table 1:**
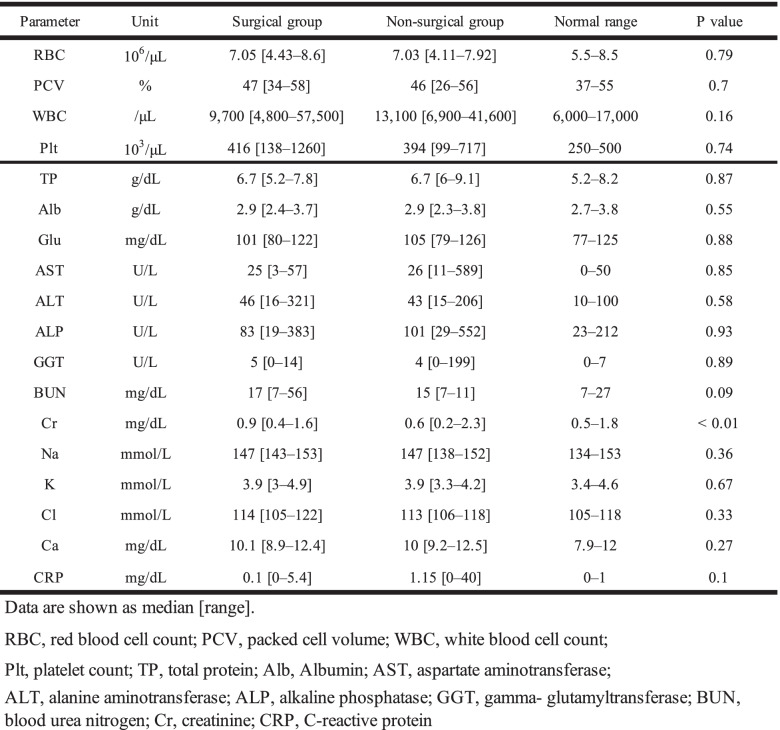
Hematology and serum chemistry examinations between the surgical and non-surgical groups

Abdominal radiographic and ultrasonographic examinations demonstrated enlargement of the prostate in all dogs. Preoperative computed tomography (CT) in the initial evaluation was performed in 29 (100%) and 10 (83%) patients in the surgical and non-surgical groups, respectively. On CT images, intra- and extracapsular (smooth and irregular) prostate masses were found in 16 (55%) and 13 (45%) patients in the surgical group, and in 7 (70%) and 3 (30%) patients in the non-surgical group, respectively. The intra- and extracapsular (smooth and irregular) types showed no significant differences between the surgical and non-surgical groups. In addition, presumed pulmonary metastases were detected in 10 (34%) and 2 (20%) patients in the surgical and non-surgical groups, respectively. Enlarged sublumbar lymph nodes were detected in 10 (34%) and 7 (63%) patients in the surgical and non-surgical groups, respectively. The detection status of the lung and lymph nodes was not significantly different between the surgical and non-surgical groups. In addition, no evidence of suspicious of bone metastasis was observed. Invasion into the urethra/urinary bladder was assessed in 31 cases (21 surgical cases and 10 non-surgical cases) by CT. Invasion of the urethra and urinary bladder was found in 11 (52%) and 15 (62%) patients in the surgical group, respectively, and in 8 (80%) and 8 (80%) patients in the non-surgical group, respectively. There was no significant difference between the surgical and non-surgical groups for invasion of the urethra and urinary bladder (urethra, *P* = 0.140; urinary bladder, *P* = 0.935). The prostatic volume derived from the CT images was measurable in 28 patients (19 and 9 patients in the surgical and non-surgical groups, respectively). The prostatic volume and its index were 19.09 mL [4.54–42.85 mL] and 2.93 mL/kg [0.53–9.32 mL/kg], respectively. The prostatic volume in the surgical group (18.68 mL [4.54–38.92 mL]) was not significantly different from that in the non-surgical group (22.82 mL [7.79–42.85 mL]) (*P* = 0.333). Likewise, the prostatic volume index in the surgical group (2.72 mL/kg [0.53–5.78 mL/kg]) was also not significantly different from that in the non-surgical group (3.46 mL/kg [1.69–9.32 mL/kg]) (*P* = 0.360).

In all surgical cases, the histopathological diagnosis was prostatic adenocarcinoma. Dirty margins were observed in 13 cases (13/29 cases, 44%); otherwise, clear margins were observed in 16 cases (16/29 cases, 55%). Margin evaluations were not significantly different between the TP and TPC subgroups. The MST of cases with dirty margins (13/29 cases) was 262 days after the initial evaluation and 253 days after surgery. The MST of cases with clean margins (16/29 cases) was 443 days after the initial evaluation and 416.5 days after surgery. The MST was not significantly different between margin types (MST from initial evaluation; *P* = 0.488, MST after surgery; *P* = 0.476).

In the non-surgical group, NSAIDs were orally administered to 13 patients. Carprofen (5.7 mg/kg, SID), firocoxib (4.3–9.3 mg/kg, SID), meloxicam (0.1 mg/kg, SID), and piroxicam (0.3 mg/kg, every other day) were used in 1, 9, 1, and 2 patients, respectively. In addition, toceranib (2.2 mg/kg, EOD) was prescribed in 1 patient. The NSAIDs and toceranib were continued as long as the patient tolerated the oral medication.

In the surgical group, oral NSAIDs administration was performed in 27 patients for postoperative adjuvant therapy: carprofen (3.8–4.3 mg/kg, SID), firocoxib (4.3–9.8 mg/kg, SID), meloxicam (0.07–0.11 mg/kg, SID), and piroxicam (0.3 mg/kg, EOD) were prescribed in 15, 21, 4, and 2 patients, respectively. In the 17 patients undergoing postoperative chemotherapy, toceranib, carboplatin, 5-fluorouracil, and chlorambucil were used in 8, 5, 2, and 2 patients, respectively. Toceranib was administered at the dose of 1.18–2.54 mg/kg, EOD. The NSAIDs and toceranib were continued as long as the patient tolerated the oral medication. Of the remaining 9 patients, 4 underwent bisphosphonate (zoledronic acid) and adoptive immunochemotherapy (lymphocyte activated killer cells [LAK] therapy and alternative administration with low doses of carboplatin and 5-fluorouracil) in 1 and 3 patients, respectively.

**Fig. 10:**
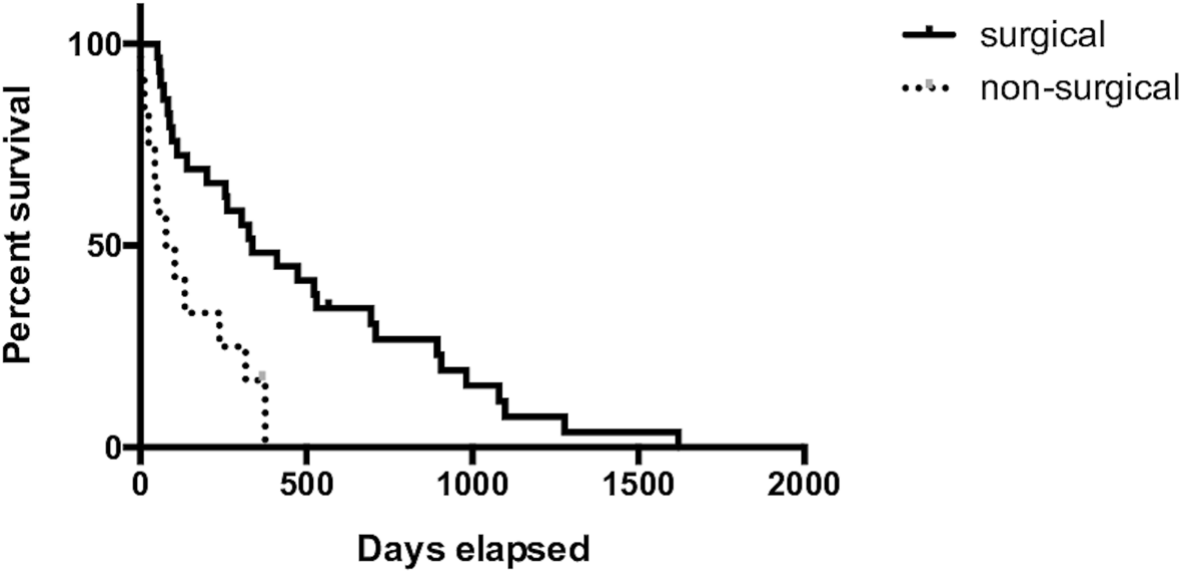
Kaplan–Meier curves of the surgical and non-surgical group mean survival time (MST) from the initial evaluation. The MST in the surgical group was significantly longer than that in the non-surgical group (337 vs. 90.5 days; *P* = 0.002)

The overall MST from the initial evaluation was 262 days (2–1620 days). MST from the initial evaluation was 337 (52–1620 days) and 90.5 days (2–369 days) in the surgical and non-surgical groups, respectively. The survival time in the surgical group was significantly longer than that in the non-surgical group (*P* = 0.002; Fig. 10). Furthermore, when comparing patients with extracapsular and intracapsular invasion depicted by CT, MST (107.5 days) from the initial evaluation in cases with (irregular) extracapsular invasion was significantly shorter than that (337 days) in cases with (smooth) intracapsular invasion. (*P* = 0.034). Comparison of patients with small pulmonary nodules on CT demonstrated that the survival time from the initial evaluation showed no significant difference between the surgical (103.5 days [52–906 days]) and non-surgical groups (134 days [24–317 days]). Additionally, comparison of patients with enlarged sublumbar lymph nodes in the CT findings demonstrated that the survival time from the initial evaluation showed no significant difference between the surgical (118 days [52–1080 days]) and non-surgical groups (238 days [2–376 days]).

**Fig. 11:**
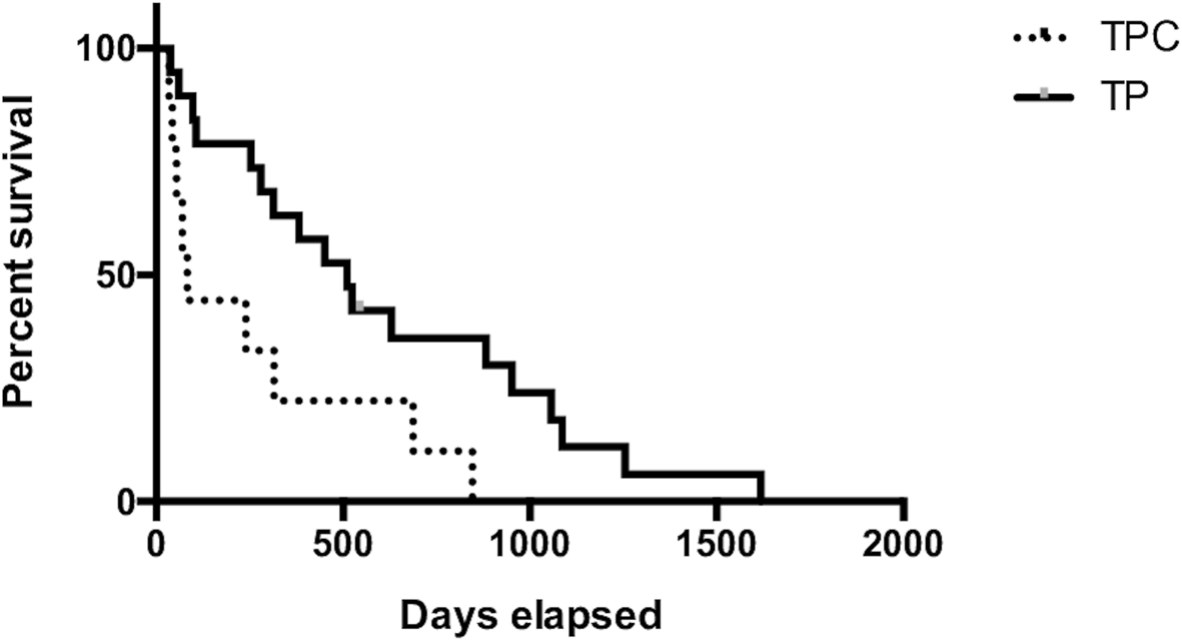
Kaplan–Meier survival curve of each surgical group after the operation. The mean survival time (MST) in the TP group was significantly longer than that in the TPC group (510 vs. 83 days; *P* = 0.017)

Of the 29 patients in the surgical group, 20 and 9 patients underwent TP and TPC, respectively. In only 1 patient in the TP subgroup, local recurrence was observed at 4 months after the first surgery (TP), and we performed a second operation with total cystectomy (TC) in which each ureter was anastomosed individually to the prepuce. In this patient, the survival time from the initial evaluation was 201 days, and survival from the first and second surgeries were 191 days and 86 days, respectively. This patient was excluded from the comparison of survival time between TP and TPC. The MST from the initial evaluation was 523 days (60–1620 days) and 96 days (52–894 days) in the TP and TPC subgroups, respectively. The survival time was significantly longer in the TP group than in the TPC subgroup (*P* = 0.013). The postoperative MSTs were 510 (37–1618 days) and 83 days (34–846 days) in the TP and TPC subgroups, respectively. The postoperative survival time was significantly different between the TP and TPC subgroups (Fig. 11) (*P* = 0.017).

In terms of postoperative complications, urinary incontinence was observed in 16 patients (16/20 cases; 80%) in the TP subgroup (non, mild and severe in 4 [20%] 10 [50%] and 6 patients [30%], respectively). Mild cases had intermittent incontinence with voluntary micturition. Six patients with severe incontinence and all patients in the TPC subgroup had constant urinary dribbling. However, even in these cases, urinary incontinence was easily managed with diapers. In addition, postoperative pyelonephritis occurred in 3 patients in the TPC subgroup (30%) but no patients in the TP subgroup.

## Discussion

Previous studies have reported that the MSTs of dogs with prostatic cancer treated using NSAIDs and those remaining untreated were 6.9 and 0.7 months, respectively [7]. Furthermore, the MST of dogs with prostatic carcinomas treated with only NSAIDs and those treated with NSAIDs and chemotherapy were 51 and 106 days, respectively [9]. In our study, the MST of the dogs treated with NSAIDs was 90.5 days, which was shorter than that in previous reports [7, 9], which was attributed to differences in breeds, histopathological type, and tumor progression. Currently, the consensus derived from previous clinical studies is that the impact of surgery and radiation on the survival of dogs with prostatic cancer is limited compared with medical treatment [7, 14, 20]. A previous report on the treatment of canine prostatic cancers demonstrated that subtotal intracapsular prostatectomy was superior to TP in terms of postoperative survival and serious complications [6]. However, no surgical interventions have been conclusively demonstrated to hold the potential to improve survival time in dogs with prostatic cancers [14, 21–24]. In our study, MST was longer in the surgical group than in the non-surgical group (337 vs. 77 days). However, the patients with either pulmonary metastasis and/or sublumbar lymphadenopathy in the surgical group had similar survival time to those in the non-surgical group. Although postoperative adjuvant therapy, such as NSAID administration and/or chemotherapy, was performed in all cases, surgical treatment may provide more survival benefits than medical therapy. However, in case where metastasis is suspected, surgical intervention and/or NSAIDs administration may not have the survival benefit.

Regarding TP, the MSTs for canine prostatic carcinoma and adenocarcinoma were reported to be 231 and 248 days, respectively [15]. However, in our study, MST in the surgical group was 337 days after the initial evaluation. Therefore, the survival time was significantly higher in the surgical group than in the non-surgical group and in previous studies [14, 15]. Moreover, regarding the postoperative MST, TP had a longer life-prolongation effect than TPC (510 vs. 137 days). In our study, the patients in the TPC subgroup might have more advanced disease compared with those in the TP subgroup. In humans, the infection incidence rates after total cystectomy are reported to be 9–19.2% [25, 26]. A few reports have suggested that the occurrence rate of pyelonephritis after total cystectomy is 5/19 and 3/19 kidneys, respectively [18, 19]. In our study, pyelonephritis was suspected to be present in 3 of 10 dogs in the TPC subgroup. The risk of developing pyelonephritis was higher in the TPC group than in the TP group. The MST of tumor progression and postoperative pyelonephritis was shorter after TPC than after TP. To improve prognosis, a definitive diagnosis of prostatic cancer at an early stage and early surgical intervention is considered very important.

Both surgeons and pet owners are concerned about postoperative complications in these animals. Particularly, postoperative urinary incontinence is thought to be a serious issue [11–14]. There is no optimal urinary incontinence scoring for postoperative complications; this often involves subjective scoring. Therefore, a 3-level scoring system was utilized in this study. Clinical signs related to prostatic adenocarcinoma were resolved postoperatively in all our cases. TP caused mild or no incontinence in most cases, while voluntary micturition was maintained in 68.4%. However, severe incontinence was observed after TP in 31.6% of cases; this incidence rate was approximately the same as in a previous report (34.8%) [15]. Therefore, early diagnosis of prostatic adenocarcinoma was particularly important for allowing TP to be performed at an early stage. For the early detection, routine rectal examination, abdominal ultrasound and urinary BRAF genetic examination might be effective. Further investigations are needed for clarify whether these procedures serve as an early detection of canine prostatic adenocarcinomas.

The previous study has not yet demonstrated a significant difference of survival time between clear and dirty margins [15]. In our study, the MST was not significantly different between margin types. However, the patients with clean margin tended to have a longer survival time than those with dirty margin. Therefore, the larger sample size may produce the significant difference between the surgical margin type. Further investigations using larger number of surgical cases in canine prostatic adenocarcinoma are needed to clarify the association between surgical margin type and prognosis.

Our study had some limitations, as there were differences in dog breeds (small-breed dogs) between Japan and other countries, there was a lack of accurate information regarding postoperative urinary incontinence, and variation in medical therapy and surgical procedures. This was a retrospective study in which the owners selected medical or surgical treatment for their pets. To resolve these limitations, randomization of surgical or non-surgical procedure selection and universal objective evaluation of urinary incontinence are required. Moreover, in our study, different treatment groups could not be evaluated for stratification as there is not a well-defined staging scheme and well-defined prognostic factors for prostatic adenocarcinomas in dogs.

## Conclusion

Results of the study suggest that surgical treatment of prostatic carcinoma results in longer survival times over medical management alone. In particular, TP might be recommended for improving survival time and quality of life in canine prostatic adenocarcinoma that does not infiltrate the bladder. Early detection is key for a survival advantage with surgical treatment.

## Data Availability

All data supporting the conclusions of this article are included within the article.

## Abbreviations

CT: Computed tomography
MST: Median survival time
QOL: Quality of life
TC: Total cystectomy
TP: Total prostatectomy
TPC: Total prostatocystectomy
